# Exploring Perception Types of Humanities Job Seekers in Employment Preparation: Implications for Career Guidance

**DOI:** 10.3390/bs15020151

**Published:** 2025-01-30

**Authors:** Je Hwa Jang, Song Yi Lee

**Affiliations:** Department of Counselling and Coaching, Dongguk University-Seoul, 30, Pildong-ro 1 gil, Jung-gu, Seoul 04620, Republic of Korea

**Keywords:** humanities majors, employment preparation, perception types, Q methodology, social support

## Abstract

This study uses Q methodology to examine the perception types of humanities majors during their employment preparation process. With the rapid advancement of artificial intelligence (AI) and automation technologies, traditional career paths for humanities majors are shrinking, leading to intensified job mismatches, psychological anxiety, and social bias. The study identified four perception types: (1) Social Support for Career Challenges, which emphasises the need for emotional and institutional support to overcome career-related anxiety and biases, (2) Building Practical Career Skills, which focuses on enhancing employability through practical job experience and technical skill development, (3) Graduation-related Career Constraints, which highlights the limitations caused by academic graduation requirements, calling for structural reforms and expanded certification support, and (4) Proactive Job Preparation, which reflects active efforts to adapt to technological advancements and competitive job market demands by emphasising digital skill acquisition and practical education. We analyse each type’s characteristics and support needs, offering valuable insights into how to address these challenges. The findings provide policy implications for career guidance and employment support, aiming to improve the employment success rates and job stability of humanities graduates. By offering empirical evidence for tailored support programmes, this study contributes practical recommendations to prepare humanities majors for the evolving job market.

## 1. Introduction

Recent advancements in artificial intelligence (AI) and automation technologies have significantly reduced traditional career paths for humanities majors, limiting employment opportunities in their fields of study and intensifying competition in the job market. Data from the Korea Employment Information Service (2024) highlights a contrasting trend: industries such as chemical and environmental installation and maintenance, metal and materials installation and maintenance, and production are experiencing labour shortages. Meanwhile, job seekers pursuing professions traditionally associated with humanities majors—such as arts, design, broadcasting, management (executives, department heads), business administration, and administrative office positions—are facing significant challenges in securing employment (Korea Employment Information Service, 2024).

In South Korea, the process of securing a first job for university graduates involves highly competitive recruitment procedures. Many graduates are in the early stages of entering the labour market, requiring careful and rational decision-making when selecting their initial career paths (D. B. Jeong, 2021). Academic achievements, professional certifications, and extracurricular activities serve as key factors in enhancing employability. Job seekers often participate in recruitment programmes organised by companies or job fairs hosted by universities, while internship experiences and networking within industries play a critical role in securing entry-level positions (J. Y. Yoon & Lim, 2020). These recruitment practices highlight the significance of academic and professional excellence as essential criteria for evaluating employability in South Korea’s cultural and economic context.

Blustein (1989) emphasised the importance of identifying one’s intrinsic characteristics and gathering and analysing specific information required by the professional world to find a suitable job. This process is particularly critical for humanities majors who face greater challenges in career and employment (H. B. Jang & Park, 2018). Consequently, humanities majors often struggle with defining clear career directions and ensuring job stability. While many young job seekers encounter various problems and anxieties during the career selection and preparation process, their perceptions are highly individualistic and complex (K. J. Park & Kim, 2024). The employment difficulties of humanities graduates are not solely due to personal career issues. Therefore, we need comprehensive research that considers the social and environmental factors affecting their employment (Y. Kim & Lee, 2023).

These technological changes do more than transform job structures; they exacerbate psychological anxiety and confusion caused by job mismatches. In particular, the rapid advancement of AI and automation technologies increases uncertainty in the career decision-making process for university students, which may negatively impact career anxiety and life satisfaction (Parkin, 2024). However, studies indicate that psychological support and efforts to enhance self-efficacy can help alleviate this anxiety among university students (Müceldili et al., 2023).

For humanities majors, additional challenges arise from time constraints related to fulfilling graduation requirements and the pressure to enhance employment competitiveness through “spec-building”, a practice widely recognised in South Korea. “Spec-building” encompasses employment preparation behaviours such as obtaining various certifications, participating in internships, studying abroad for language training, and gathering information about future careers. It is a means of packaging one’s value in the labour market. Job seekers restructure their experiences to align with the logic of the labour market through spec-building, recognising it as an essential requirement to improve their employability (S. Y. Jang, 2013).

According to Frey and Osborne (2017), automation technologies have the potential to replace tasks involving simple cognition and repetitive work, significantly affecting certain professions traditionally pursued by humanities and social sciences majors. They estimate that 47% of jobs are susceptible to automation, and even professions requiring creativity and critical thinking are at risk of partial displacement. This finding underscores the increasing limitations in employment opportunities for humanities majors.

Arntz et al. (2016) highlight that advancements in AI and automation technologies increase the labour market pressures on highly educated humanities and social sciences graduates. They noted that automation necessitates a reassessment of humanities-related skills and competencies, even in roles with low technological substitutability. This dynamic further exacerbates employment insecurity for humanities and social sciences majors. Additionally, their study analysed the specific challenges faced by graduates who struggle to adapt to these technological advancements, shedding light on the difficulties of maintaining relevance in a rapidly evolving job market.

However, technological changes are not the only challenges humanities majors face; psychological and environmental factors also have significant influence. These include career anxiety, the competitive pressure to build credentials, time constraints from graduation requirements, and the mismatch between their academic specialisation and job roles (H. B. Jang & Park, 2018). While many students engage in various activities to overcome their psychological anxiety, there is a pressing need for systematic career counselling and psychological support (Sun et al., 2012).

Moreover, humanities majors often experience a disconnect between their theoretical, discipline-focused education and the practical experience required in the workforce. This gap highlights the importance of providing employment support through practical, skills-based training programmes (Won, 2019). Additionally, graduating students often struggle with the time constraints of meeting graduation requirements while preparing for employment, facing intense competition and feeling the need for advanced degrees or additional skill development (Shin, 2016). Systematic analysis of these issues is essential to support humanities majors during their employment preparation process.

Humanities majors, unlike disciplines such as medicine, arts, education, or engineering, connect less directly to specific career fields and include a broader range of career exploration (J. S. Jeong et al., 2011). This lack of clear alignment with job roles often leads to feelings of uncertainty and anxiety during the career decision-making process. Additionally, the burden of meeting graduation requirements and the pressure of competing in credential-building further exacerbate the psychological and environmental challenges faced by humanities students, complicating their employment preparation journey.

Most humanities majors, which primarily focus on pure academic disciplines, often struggle to adapt to the rapidly advancing technological innovations and increasing complexity of modern industrial environments (Y. Kim & Lee, 2023). In particular, there is a need for research on how humanities majors evaluate their academic knowledge and skills and use them to make specific career choices and define their career paths. Humanities graduates tend to experience lower alignment between their academic specialisation and job roles compared to other fields, and they also exhibit relatively shorter job retention periods (J. Y. Jeong et al., 2018).

In this context, it is essential to examine the subjective perceptions of young humanities job seekers regarding key barriers in the employment preparation process, including motivational factors, psychological anxiety, and social support. Analysing these perceptions provides valuable insights into the practical challenges faced by humanities graduates and helps in designing effective support measures to enhance their employment preparation experiences (Korea LaborLabour Institute, 2019). Identifying and categorising these perceptions, along with the anxiety factors related to career choice and preparation, is a crucial starting point for addressing their career challenges and guiding them towards successful outcomes.

By categorising perceptions into distinct types, we can systematically classify various challenges and anxiety factors and identify specific difficulties and support needs for each type (H. Y. Jang & Lee, 2019). Such categorisation helps to understand the psychological and environmental factors influencing job seekers and provides a foundation for more effective career guidance and tailored support strategies. Through this process, humanities majors can experience a more positive employment preparation journey. Additionally, it offers actionable directions for social and institutional interventions, such as psychological support, the development of practical education programmes, and institutional measures to alleviate the pressure of competitive credential-building (J. S. Jeon & Oh, 2023).

Therefore, this study classifies the perception types of young humanities job seekers regarding their employment preparation process and identifies the specific challenges and support needs associated with each type. Through this, the study seeks to propose specialised career guidance and employment support measures tailored to humanities majors, ultimately contributing to enhancing their job stability and improving their employment success rate. Our research questions (RQ) are as follows:

RQ1. How can we categorise the perception types of young humanities job seekers regarding their employment preparation process?

RQ2. What are the characteristics of each perception type regarding the employment preparation process for young humanities job seekers?

## 2. Materials and Methods

### 2.1. Q Methodology Research Procedure

William Stephenson (1953), the founder of Q methodology, described it as a scientific approach to exploring individual subjectivity. He argued that Q methodology enables a quantitative expression of thoughts, feelings, and perceptions. Specifically, Stephenson highlighted its value in systematically analysing participants’ subjective views and classifying them into various perception types. Expanding on this, Brown (1980) defined Q methodology as a unique approach that integrates qualitative and quantitative research methods, making it an effective tool for studying subjective perspectives.

Q methodology is critical in analysing complex social and psychological phenomena by scientifically exploring and classifying individual subjectivity. Brown (2009) emphasised its multidisciplinary applications, particularly its ability to measure subjective perceptions and track changes over time. In essence, Q methodology investigates participants’ internal cognitive structures by posing questions on specific topics and systematically analysing subjective perspectives, which can vary depending on abstract or individual characteristics.

**Figure 1:**
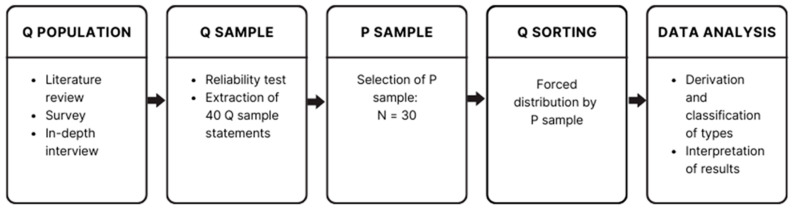
Research procedure of Q methodology.

This approach is especially effective for objectively and practically studying diverse viewpoints, providing a deeper understanding of unique individual perspectives (Y. C. Kim & Lee, 2020). Its flexibility and precision make it an indispensable tool for researchers across various fields. The research procedure for Q methodology comprises five steps: constructing the Q population, selecting the Q sample, selecting the P sample, Q sorting and data analysis. Figure 1 illustrates these steps (Brown, 1980).

### 2.2. Constructing the Q Population

The Q population is a collection of statements that represent diverse subjective perspectives on a specific topic. These statements come from various sources, such as literature reviews and interviews, to ensure a comprehensive and representative collection of viewpoints. The statements are self-referential and effectively capture individual opinions (Y. C. Kim & Lee, 2020).

As the foundational material for exploring diverse thoughts and opinions, the Q population is the basis for selecting the final Q sample. H. G. Kim (2008) defines the Q population as a set of statements encompassing all stimulus items related to a specific topic, constructed by the researcher through various approaches like literature reviews, surveys, and in-depth interviews to reflect a wide range of opinions and perspectives. By encompassing diverse viewpoints, the Q population provides a critical starting point for systematically analysing participants’ subjective perceptions. In this study, we developed the Q population using a combination of literature analysis, semi-structured survey questionnaires, and in-depth interviews.

First, we reviewed domestic articles, academic journals, and books related to the employment of young humanities majors. This process resulted in 73 statements, such as “Feeling burdened to consider pursuing additional academic degrees to enhance employment prospects” (H. B. Jang & Park, 2018).

Second, we used semi-structured questionnaires to conduct in-depth interviews. Sample questions included:

- How do you perceive the impact of a humanities major on employment?
- What challenges do humanities majors commonly encounter during employment preparation?
- How do advancements in AI and automation technologies influence the employment preparation of humanities majors?
- What kinds of support or assistance do you think are necessary during the employment preparation process?

Interview participants were from five humanities-related departments at A University in Gyeonggi Province. Through this process, we gathered 74 statements, bringing the final Q population to 147 statements.

### 2.3. Selecting the Q Sample

The Q sample is a carefully selected set of statements that represent the study’s core themes. According to Brown (1980), the Q sample comprises statements that reflect a comprehensive range of perspectives and attitudes related to the research topic. Its primary goal is to capture representative statements from the broader Q population, effectively encapsulating the study’s main themes. The selection process is critical in Q methodology, as it significantly impacts the research outcomes (H. G. Kim, 2008).

For this study, we determined the Q sample to be between 40 and 60 statements, following established guidelines (H. G. Kim, 2008). We constructed the Q sample by carefully reviewing the Q population multiple times to identify statements that aligned with the research topic. We removed ambiguous, redundant, or difficult-to-understand expressions to ensure clarity and precision in the final set of statements.

Subsequently, we conducted a preliminary test with four research participants from A University to evaluate the statements’ readability and comprehensibility; we made final revisions based on their feedback. This process ensured that the Q sample sufficiently reflected the diverse perspectives and attitudes of humanities majors regarding employment and career paths. Additionally, four experts familiar with Q methodology reviewed and confirmed the final selection of 40 statements for the Q sample. Table 1 presents the final Q sample statements.

### 2.4. Selecting the P Sample

The P sample refers to the study’s participants selected from the initial Q population group based on shared experiences, beliefs, or interests (S. E. Kim, 2016). In essence, the P sample represents the individuals participating in the research. The selection process ensures the group includes diverse perspectives on the research topic.

According to H. G. Kim (2008), Q methodology does not require a large sample size; in fact, too many participants can lead to responses clustering around specific factors, which may obscure distinct characteristics. Unlike R methodology, Q methodology typically uses smaller sample sizes, with an appropriate range between 20 and 60 participants. Thus, this study’s P sample comprises 30 participants, including fourth-year students in their final semester and recent graduates as of October 2024 from A University in Gyeonggi Province. The participants were from five departments: Korean Language and Literature, English Language and Culture, Chinese Language and Culture, Russian Language and Culture, and Early Childhood Education. The sample comprised 10 male and 20 female participants. We ensured equal representation by selecting six participants from each department within the College of Humanities.

### 2.5. Q Sorting

Q sorting is the process in which research participants (the P sample) arrange the given statements (the Q sample) based on their subjective perspectives. Rather than simply evaluating the statements, participants rank them on a continuum ranging from “agree” to “disagree” based on their beliefs, experiences, or attitudes. This process follows a specific distribution, such as a normal distribution, predetermined by the researcher, requiring participants to sort the statements into a forced distribution. By doing so, Q sorting systematically captures and explores participants’ subjective viewpoints. As a core technique of Q methodology, it is essential for uncovering and revealing participants’ perspectives (Watts & Stenner, 2005).

The forced distribution method requires participants to sort statements into a predetermined distribution, systematically ranking each statement according to their level of agreement. This process quantifies each participant’s unique perceptions and allows researchers to identify differences in perceptions during data analysis. This study used a scale ranging from −5 to +5 to highlight the statements with which participants most agreed and disagreed. This approach is particularly useful for capturing the intensity of subjective perceptions in greater detail. Additionally, the forced distribution method in Q sorting enables participants to express their perspectives and provides the foundation for factor analysis, which identifies the major perception types among participants (J. H. Yoon et al., 2024; J. S. Kim et al., 2023). This analysis provides a structured understanding of humanities job seekers’ challenges and needs during their employment preparation process.

**Figure 2:**
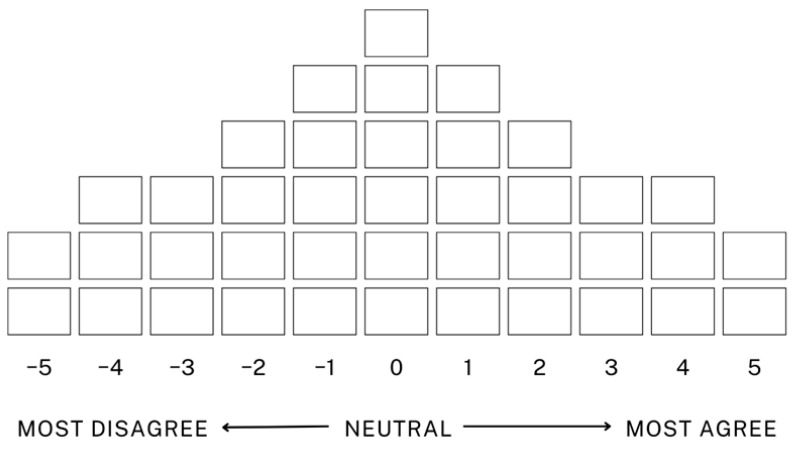
Sample sorting distribution chart.

During the Q sorting phase of this study, participants reviewed 40 statement cards, compared their content, and sorted them based on the degree of agreement or disagreement. Participants also explained their reasons for selecting the statements with which they most strongly agreed and disagreed. We analysed the data collected from the 30 participants by ranking each statement according to its degree of agreement. Figure 2 illustrates this study’s Q distribution chart.

### 2.6. Data Analysis

The study used the Ken-Q programme for PC (version 2.0.1) to conduct data analysis, combining principal component factor analysis (PCA) with varimax rotation as the rotation technique. Additionally, we analysed the data using KADE (Ken-Q Analysis Desktop Edition), a cutting-edge tool that offers stability and efficiency in data processing. KADE functions as an independent desktop application, enabling quick and accurate analysis without requiring an internet connection. This approach streamlined the research process and enhanced the reliability and validity of the results.

We selected statements that best represented each factor’s characteristics, using Z-scores (standard scores) and Q-sort values to identify those associated with each factor. We interpreted these statements to define the unique characteristics of each type. Additionally, we used the explanations provided by participants with high factor loadings for their most agreed upon and most disagreed upon statements as critical data for interpreting each type’s features.

## 3. Research Results

### 3.1. Q Factor Analysis Results

We derived the factor classification results using an eigenvalue threshold of 1.0 or higher. The eigenvalues for the four identified factors were as follows: Type 1 = 8.82696567, Type 2 = 4.44859718, Type 3 = 2.3917349, and Type 4 = 1.90183949. The cumulative variance analysis showed that Types 1 through 4 collectively explained 58% of the total variance. This result demonstrates that the Q-analysis methodology used in this study effectively accounts for approximately 58% of the variance in the diverse perception types that humanities majors hold regarding the employment preparation process. Table 2 outlines the eigenvalues and explanatory variables of the four types.

The correlation between each type indicates the similarities among them. As shown in Table 3, the correlation values are as follows: Type 1 and Type 2 = 0.1712, Type 1 and Type 3 = 0.0916, Type 1 and Type 4 = 0.6305, Type 2 and Type 3 = 0.2534, Type 2 and Type 4 = 0.1252, and Type 3 and Type 4 = 0.2244.

### 3.2. Standard Scores (Z-Scores) and Q-Sort Values for Q Statements by Type

We categorised the standard scores (Z-scores) and Q-sort values for Q statements by type (refer to Table 1). A positive (+) Z-score indicates strong agreement with the statement, while a negative (−) Z-score indicates strong disagreement. Larger absolute values reflect a stronger intensity of agreement or disagreement. By analysing the Z-scores for each type, we can identify the unique characteristics of each group and gain insights into the subjective perceptions of the P-sample participants within those types.

### 3.3. Factor Loadings by Type

Table 4 shows the demographic characteristics of the study participants and their factor loadings by type. The analysis of 30 participants resulted in four independent types. Specifically, we identified ten participants as Type 1, six as Type 2, three as Type 3, and eleven as Type 4. We considered participants with high factor loadings within each type as representative of that type.

### 3.4. Characteristics by Type

This study identified four perception types among humanities students during their employment preparation process.

Type I, Social Support for Career Challenges, emphasises the importance of social and emotional support in overcoming career-related anxiety and biases. It also highlights the need for practical experience and institutional backing to navigate professional challenges effectively.

Type II, Building Practical Career Skills, reflects a tendency to enhance employability through practical job experience and skill development, emphasising the value of real-world practice in career preparation.

Type III, Graduation-related Career Constraints, highlights the limitations imposed by academic graduation requirements, advocating for more flexible policies and expanded certification support programmes to ease the transition into the workforce.

Finally, Type IV, Proactive Job Preparation, highlights proactive efforts to address the pressures of credential requirements and technological advancements. It emphasises the importance of acquiring digital skills and engaging in practical educational programmes to stay competitive in the job market.

#### 3.4.1. Type I: Social Support for Career Challenges

The key statements with which those in Type I (Social Support for Career Challenges) strongly agreed relate to prejudice and the need for improved perception (Statement 35), career anxiety (Statement 39), emotional support from family (Statement 2), and difficulties in career decision-making (Statement 37). Type I reflects concerns about prejudice and anxiety related to career paths and highlights the importance of emotional support. Additionally, this type expressed a desire to address career anxiety by emphasising opportunities for practical experience (Statement 22), lectures by professionals in the field (Statement 7), and activation of job-related systems (Statement 9).

The participants in the Social Support for Career Challenges type disagreed with several statements, including graduation requirements interfering with job preparation (Statement 38), lack of time for career development (Statement 16), difficulties in choosing double or minor majors (Statement 19), denial of technical advantages of language skills (Statement 4), and limited job-related information on websites (Statement 10). Notably, Types III (Graduation-related Career Constraints) and IV (Proactive Job Preparation) also generally disagreed with Statement 10. A key distinction for Type I is their strong agreement with Statement 35, reinforcing the need to address prejudice and improve societal perceptions of humanities majors. In contrast, participants from other types consistently maintained a neutral stance on this issue.

P28, representing Type I with the highest factor loading, stated, “Due to prejudice against the Korean Language and Literature major and repeated questions about employment, I feel less confident and intimidated as I approach graduation”. Similarly, P12 remarked, “There has always been a significant difference in perception and employment rates between humanities and STEM fields, and prejudice against humanities majors, especially language-related majors, seems particularly severe”. Most participants expressed difficulties with making career choices that align with their strengths. They also highlighted the need for opportunities to gain practical experience and the importance of taking active steps to address these challenges.

Therefore, the Social Support for Career Challenges type requires practical experience and institutional support to overcome career anxiety and uncertainty, with a particular emphasis on the importance of enhancing technical skills and providing information. However, economic burdens and academic demands pose additional obstacles to their employment preparation. This type demonstrates a proactive attitude towards overcoming career anxiety through practical efforts and strongly advocates for reducing societal prejudice and expanding institutional support.

#### 3.4.2. Type II: Building Practical Career Skills

This group (the Building Practical Career Skills type) mostly strongly agreed with statements focusing on the importance of mentoring that connects real-world job experience to employment (Statement 32) and practical education applicable in the workplace after employment (Statement 21), reflecting their demand for targeted, practical support. They also strongly agreed with statements about difficulties in selecting double or minor majors due to the demands of their primary field (Statement 19) and the lack of opportunities and information for entering fields unrelated to their major (Statement 17).

These views highlight challenges not in the connection between their major and career but in the nature of the Early Childhood Education major. This field involves a heavy course load and mandatory certification, leaving little free time. As P18 noted, “Due to the nature of the Early Childhood Education major, there are many mandatory courses required to obtain certifications, leading to both mental and physical exhaustion, making it burdensome”.

Additionally, this type expressed a strong need for support in expanding digital education related to the AI industry (Statement 23), institutional support for learning IT skills (Statement 27), and providing information about various certifications and acquisition processes (Statement 28). These responses underscore a desire to enhance technical skills and improve access to career-related information.

This type disagreed with or expressed neutrality towards several statements, including foreign language skills not being a technical strength in the workplace (Statement 4), anxiety about uncertainty regarding career paths after graduation (Statement 39), difficulty expressing one’s competencies in a portfolio (Statement 15), difficulty in identifying strengths or talents when choosing a career (Statement 37), and difficulty with graduation requirements (Statement 38).

P16 and P23 stressed the value of foreign language skills, stating, “In a multicultural and global society, foreign language skills are one of the greatest strengths and should be developed alongside professional competencies”.

The Building Practical Career Skills type stands out among humanities majors due to its unique characteristics. Unlike Types I (Social Support for Career Challenges), III (Graduation-related Career Constraints), and IV (Proactive Job Preparation), as well as students from other departments, who largely agreed with Statement 15 (“It is difficult to express one’s competencies in a portfolio”), students majoring in Early Childhood Education were more likely to disagree or remain neutral. P17 explained, “Due to the nature of the major, it is closer to a professional field, and there is high self-efficacy regarding the job role, with clear career paths after graduation”. This response contrasts sharply with the anxiety about career uncertainty reflected in Statement 39.

Participants in this type prioritise enhancing career competencies through practical and specific support. This group demonstrates a pronounced demand for developing technical skills, expanding job experience opportunities, and improving access to information, all while underscoring the importance of emotional support and mentoring. In contrast, they perceive economic burdens and abstract anxieties as relatively less significant. The core characteristic of this type is its focus on practical, solution-oriented approaches.

#### 3.4.3. Type III: Graduation-Related Career Constraints

The Graduation-related Career Constraints type mostly agreed with statements highlighting the difficulty of focusing on employment preparation due to strict graduation requirements (Statement 38), the need to expand support for mandatory certifications for humanities majors (Statement 8), and the importance of increasing internship opportunities to gain practical experience (Statement 22). Other statements that resonated with this type included the necessity of flexible emotional support from family (Statement 2), institutional support for advanced IT education (Statement 27), and practical curriculums applicable in the workplace after employment (Statement 21).

In contrast, this group disagreed with or were neutral towards statements such as uncertainty in career choices due to an inability to identify strengths or talents (Statement 37), difficulty gathering information because of limited job-related websites (Statement 10), lack of support programmes for job exploration (Statement 6), anxiety about post-graduation career uncertainty (Statement 39), and insufficient employment competency programmes for humanities majors (Statement 30).

This type’s defining characteristic is its strong agreement with “difficulty focusing on employment preparation due to strict graduation requirements (Statement 38)”. P23 highlighted this challenge, stating, “The department requires three language certifications as part of the graduation requirements, which creates significant pressure before I can even start preparing for employment”. They further remarked, “If graduation requirements were made more flexible, such as reducing the number of certifications required or making them optional, it would be easier to focus on employment preparation”.

Another notable characteristic of this type is their demand for expanded support for mandatory certifications for humanities majors (Statement 8). Compared to Types I (Social Support for Career Challenges), II (Building Practical Career Skills), and IV (Proactive Job Preparation, participants in the Graduation-related Career Constraints type expressed a stronger need for certification-related education to support employment. Additionally, they underscored the importance of expanding internship opportunities to help humanities majors gain practical experience (Statement 22).

The statements with which Type III most disagreed or perceived negatively include uncertainty in career choices due to an inability to identify strengths (Statement 37), difficulty gathering job information due to limited resources (Statement 10), and lack of support programmes for job exploration (Statement 6).

Based on the Z-scores, concerns regarding a lack of information were not significant for this group. Instead, the primary obstacle for this type is the burden of graduation requirements, which significantly hinders employment preparation. Unless institutions implement practical support measures—such as easing graduation requirements or providing assistance with certifications—to secure more time for job preparation, students are likely to continue facing challenges in the employment process.

#### 3.4.4. Type IV: Proactive Job Preparation

The Proactive Job Preparation type strongly agreed with statements highlighting the burden of increasing demand for skills and credentials in the recruitment process (Statement 29), the pressure to consider additional academic degrees to improve employment prospects (Statement 40), the necessity of various credentials for humanities majors (Statement 31), the challenges of job seeking due to a lack of experience (Statement 13), and the need for more practical education programmes applicable immediately in the workplace after employment (Statement 21).

Conversely, participants in this type most disagreed or did not exhibit a positive perception of statements relating to difficulty in choosing double or minor majors due to the characteristics of the major (Statement 19), lack of time for career development activities due to a heavy course load (Statement 16), foreign language skills not being a technical strength in the workplace (Statement 4), the complexity and inaccessibility of social employment support systems like the national employment support programme (Statement 24), and lack of support programmes for job exploration (Statement 6).

What distinguishes the Proactive Job Preparation type from other types is their strong agreement with Statement 40 (“The pressure of considering additional academic degrees to improve employment prospects”). P27 explained, “To get a job with a major in Korean Language and Literature, a master’s degree or higher is generally required, so I am considering further academic studies”. Additionally, most Type IV participants strongly agreed with Statement 29 (“Feeling burdened by the increasing demand for skills and credentials in the recruitment process”). P2 noted, “The current trend is that the focus is on digital industries due to technological advancements, so the importance of humanities literacy and language skills is declining. I feel that I need to learn technical skills outside of my humanities major”. Participants also agreed with “Humanities majors require various credentials” (Statement 31) and “Lack of job experience makes job seeking difficult” (Statement 13), indicating a general sense of anxiety and burden regarding their credentials and work experience.

This type strongly disagreed with the statement, “It is difficult to choose a double or minor major due to the characteristics of the major” (Statement 19). P2 and P8 remarked, “As a language major, it was easy to choose a related minor or double major, and it wasn’t difficult”. This comment contrasts sharply with the experience of Type II (Building Practical Career Skills) participants, who found the graduation requirements for the Chinese language major burdensome, making it challenging to pursue a double or minor major.

Ultimately, the Proactive Job Preparation type feels the greatest burden from the increasing demands for skills and credentials in the recruitment process. They strongly stress the need for internships, practical education, and support for acquiring digital skills to prepare for and overcome these demands.

## 4. Discussion

This study classified the perception types and characteristics of university students preparing for employment, specifically focusing on humanities majors, through Q methodology. The research results revealed four distinct types:

- Type I: Social Support for Career Challenges
- Type II: Building Practical Career Skills
- Type III: Graduation-related Career Constraints
- Type IV: Proactive Job Preparation

The findings provide a nuanced understanding of the diverse perceptions and challenges faced by humanities majors during their employment preparation process.

Type I (Social Support for Career Challenges) represents individuals who address anxiety and societal perceptions through practical methods. These individuals often struggle with confidence in their career paths and perceive widespread societal biases and prejudices directed at them.

Career anxiety is common among university students, stemming from psychological pressures, fears, and concerns during the employment preparation process. These challenges frequently lead to psychological and social difficulties (So & Park, 2016). Humanities students, in particular, engage in more proactive career preparation behaviours than students in other fields. However, their efforts often do not yield results proportional to the time and energy invested (H. B. Jang & Park, 2018). This pattern can lead to employment stress, further exacerbating psychological anxiety and depression (S. Y. Park & Kim, 2021).

This analysis highlights the importance of strengthening psychological support to address societal perceptions and prejudices while enhancing career self-efficacy. Career self-efficacy—the belief in one’s ability to plan and execute effective strategies in stressful situations—is vital for fostering behavioural change (Hwang & Ko, 2015).

The statements with which Type I strongly agreed, such as Statements 35, 39, 2, and 37, all relate to psychological anxiety regarding career paths. This finding indicates the need to eliminate psychological anxiety about career paths and underscores the need to reduce psychological anxiety and, through improved career self-efficacy, enable individuals to engage confidently in practical career preparation.

Students in the Social Support for Career Change type actively seek various routes for overcoming their anxiety, indicating that this process requires more than individual effort. It also calls for strengthened social support systems and broader changes in societal perceptions to create an environment where these students can thrive. To strengthen the social support system, the University Job Plus Centers, a government initiative operated by the Ministry of Employment and Labour across more than 120 universities nationwide, provide tailored career counselling and employment preparation programmes for students experiencing career and employment anxiety—programmes such as job analysis and boot camps for specific job roles, targeting humanities majors, can enhance career decision self-efficacy by providing mentorship from industry professionals.

Additionally, offering psychological counselling services and programmes aimed at enhancing self-efficacy could help students alleviate psychological anxiety and develop a positive attitude towards career exploration. Campaigns that highlight the value of the humanities, coupled with expanded collaborations with local businesses, can further improve societal perceptions and increase employment opportunities for humanities students.

Unlike Type I, students in Type II (Building Practical Career Skills) exhibit career certainty and strong career decision self-efficacy rather than experiencing anxiety about career uncertainty. Among the six participants in this type, five were women, all enrolled in the same major and department.

While Type II differs from Type I (Social Support for Career Challenges) in terms of career anxiety, both groups share a focus on seeking practical education programmes applicable in the workplace after employment and pursuing opportunities for internships and mentoring. Type II participants noted that fulfilling major course requirements and graduation obligations left them with limited time to pursue a double major or gain practical work experience, highlighting a significant challenge in balancing academic and career preparation demands.

The Building Practical Career Skills type exhibits confidence in their career path but expresses a lack of practical experience in their major and theoretical education. Consequently, they actively seek opportunities such as internships and mentoring to enhance their professional skills. However, the demands of major courses and graduation requirements leave little time for career development activities, making it difficult to address this gap. To mitigate this issue, universities should revise their curricula to allow students to balance major courses with practical experience.

Expanding practical training programmes would allow students to develop theoretical knowledge and professional skills, aligning with Krumboltz’s emphasis on learning experiences. According to Krumboltz (2009), learning is not limited to acquiring knowledge but includes gaining practical experience through diverse life situations. Positive experiences gained in this way are critical for personal and professional growth (Y. Kim & Lee, 2023).

Additionally, most Type II participants (five out of six) are majoring in Early Childhood Education, which they view as a professional occupation. Many agreed with Statement 17, highlighting the lack of opportunities and information for entering careers outside their major. From the perspective of Krumboltz’s planned happenstance theory, this suggests how life experiences and learning can unexpectedly lead to career opportunities in other fields.

The Building Practical Career Skills type demonstrates confidence in their career paths but acknowledges a lack of practical experience and actively seeks to enhance their professional skills. Ga Hye-young (Ka & Kim, 2021) noted that fieldwork and internship experiences during university increase students’ understanding of job roles and organisations, leading to better employment outcomes. Additionally, Choi and Kang (2016) conducted a study on university students participating in the Next-Generation Leader Mentoring Programme by the Korea Scholarship Foundation. The study found that higher levels of participation in mentoring programmes significantly enhanced students’ competencies, positively influencing career progression and mentoring satisfaction.

These findings suggest the need for customised support for students in the Building Practical Career Skills type. Such support should address the constraints of graduation requirements and major coursework while providing practical, hands-on training programmes and mentoring opportunities. By offering this tailored assistance, universities can help students develop the skills and competencies needed to transition seamlessly into the workplace after graduation.

Type III (Graduation-related Career Constraints) students face significant constraints in their employment preparation process due to the burden of graduation requirements. This group primarily focuses on meeting graduation requirements, particularly language certifications, dedicating most of its time to obtaining these qualifications. As a result, this type feels it lacks sufficient time to prepare adequately for employment.

Participants in the Type III group expressed a need for economic and institutional support to acquire essential qualifications and expanded opportunities to learn new skills. Notably, despite nearing graduation, these students reported greater difficulty fulfilling graduation requirements than preparing for employment. They feel that graduation requirements align poorly with real-world job preparation, leaving them unable to focus on both simultaneously. This misalignment creates anxiety about their limited time to prepare effectively for employment.

Employment preparation activities include managing academic credits, language training, English proficiency tests, obtaining certifications, gaining work experience, gathering job-related information, preparing for exams, expanding networks, attending job fairs, and submitting job applications (Y. J. Jeon, 2008). Most job seekers strive to turn these activities into strong credentials for their resumes—however, like Type I (Social Support for Career Challenges), if individuals in Type III (Graduation-related Career Constraints) engage in these activities while experiencing psychological anxiety and employment stress, the outcomes are unlikely to be positive.

To improve their self-efficacy, Type III students need to prioritise emotional support and develop clear career goals. For students nearing graduation, anticipating and planning for graduation requirements in advance is essential. Within this context, they should actively pursue various experiences that allow them to clarify and achieve career goals. Inspired by the concept of planned happenstance, students should recognise that academic experiences might unexpectedly lead to career opportunities. By adopting a proactive approach, they can better explore and take advantage of these possibilities.

Type IV (Proactive Job Preparation) students, particularly those nearing graduation, show a strong determination to succeed in the job market by enhancing their competencies. The intense pressure of the credentials competition fuels this drive, which they recognise as a critical factor in the recruitment process.

This type significantly stresses gaining job-related experience, pursuing further education, and even considering additional academic degrees to remain competitive. They strongly agreed with statements highlighting the burden of high credential demands from employers, reflecting their awareness of the challenges humanities majors often face in this area. Consequently, they are willing to dedicate sufficient time outside their academic responsibilities to focus on skill enhancement and career development. This determination underscores their belief that humanities majors are at a disadvantage in terms of credentials, motivating them to work harder to accumulate a variety of qualifications.

While gaining various experiences to build credentials is important, it does not necessarily increase employability. H. B. Jang and Park (2018) found that engaging in career preparation behaviours promotes learning and growth. For instance, the challenging processes of career exploration, decision-making, and preparation help transform these behaviours into concrete actions, bringing individuals closer to their goals while fostering personal development.

A proactive attitude is beneficial, but students should avoid accumulating credentials unquestioningly. To carry out specific career preparation, individuals must focus on the sub-element of “information-gathering activities”, which involve understanding the job market and their capabilities (B. H. Kim, 1997). For those in the Proactive Job Preparation group (Type IV), maintaining a proactive mindset is crucial. By believing in their potential and setting clear career goals, they can take deliberate, step-by-step actions. This approach will help them create a unique, competitive set of credentials tailored to their strengths rather than just accumulating generic ones to compete with others.

## 5. Conclusions

The types identified in this study reflect humanities majors’ psychological, social, and institutional challenges, focusing on issues such as career anxiety, the need to strengthen practical skills, graduation requirements, and the competitive pressure to accumulate credentials. This framework provides a systematic understanding of the psychological stress, institutional problems, and societal demands these students encounter during their employment preparation process while also offering tailored support strategies for each type.

From an integrated perspective, the study highlights the key challenges humanities majors face as they approach graduation and proposes actionable solutions, outlining the shared responsibility of universities and society in addressing these issues. Proposed strategies include psychological counselling programmes to enhance emotional stability and career self-efficacy, flexible curricula that better connect academic majors with practical experience, and creating a recruitment culture that prioritises competencies over credentials. These measures can significantly improve students’ employability.

Additionally, continuous policy and institutional efforts are essential for ensuring students experience learning and growth throughout their employment preparation journey, helping them move closer to their career goals.

In conclusion, this study systematically analysed the issues and needs arising in the employment preparation process for humanities majors. By doing so, it contributes to establishing a foundation that enables students to balance academics and career readiness while preparing for a successful future.

## 6. Limitations

This study acknowledges several limitations and reinforces the need for follow-up research to address them. First, the research sample focused exclusively on students from A University in Gyeonggi Province, limiting the diversity of the P sample. Expanding the study to a national level would better reflect the differences in major compositions and employment environments across universities, enabling a more comprehensive understanding.

Second, the study did not fully capture the diversity of humanities majors. Since the connection between each major and the job market varies, conducting comparative studies across different majors is necessary to analyse the specific characteristics and needs of each.

Third, while this study focused on students’ subjective perceptions using the Q methodology, it did not sufficiently examine how social and institutional environments influence the employment preparation process. Future studies should integrate these factors and examine quantitative data to provide more effective policy recommendations.

By overcoming these limitations, follow-up research will deepen the understanding of challenges faced by humanities majors and offer more systematic and effective guidance for employment preparation. Ultimately, this approach aims to help students balance academics with career readiness, enabling them to design successful career paths.

## Figures and Tables

**Table 1:** Q statements with Z-scores and Q-sort values by type.

No	Statement	Factor 1		Factor 2		Factor 3		Factor 4	
		Z-Score	Q-Sort Value	Z-Score	Q-Sort Value	Z-Score	Q-Sort Value	Z-Score	Q-Sort Value
1	I feel uncertain due to a lack of information linking my major to job roles.	1.04	3 *	−1.29	−3	−0.9	−2	0.06	0 *
2	Flexible emotional support from family helps ease the job search process.	1.26	4	0.89	2	1.25	4	0.36	1
3	Majoring in a foreign language can broaden opportunities for overseas employment.	0.95	2	0.56	1	1.11	3	0.45	1
4	Foreign language skills are not a technical strength in the workplace.	−1.3	−4	−1.78	−5	0.57	1 *	−1.36	−4
5	Humanities majors can challenge themselves in various fields, but the wide range of options is confusing.	0.08	0 *	−0.64	−2	−0.88	−2	−0.8	−2
6	There is a lack of job exploration support programmes for employment.	−0.79	−2	−0.79	−2	−1.43	−4	−1.13	−3
7	Employment talks by professionals in diverse fields from humanities majors are needed.	0.97	3	0.81	2	0.4	1	−0.28	−1
8	Schools should provide more support for mandatory certifications for humanities majors.	−0.49	−1 *	0.57	1	1.65	5 *	0.35	1
9	Schools should activate systems related to job fields for humanities graduates.	0.55	1	0.95	3	0.2	1	0.85	3
10	Limited job-related websites and information make it difficult to find necessary resources.	−1.3	−4	−0.19	0*	−1.45	−5	−0.94	−3
11	There is insufficient information on salaries and benefits for desired companies.	−1.11	−3	0.29	1	−0.46	−1	−0.84	−2
12	The future of office jobs seems unstable due to the impact of AI.	−0.65	−2	−0.38	−1	−0.77	−2	−0.25	−1
13	A lack of experience in relevant job roles creates difficulties in employment.	0.62	2	0.72	2	−0.49	−1 *	1.31	4
14	It is challenging to join large companies immediately after graduation.	0.26	0	−0.95	−2 *	0.2	0	0.72	2
15	It is hard to express my competencies through a portfolio.	−0.05	0	−1.5	−4 *	−0.25	0	0.46	1
16	The number of major classes makes it difficult to allocate time for career development activities.	−1.97	−5	0.13	0	0.87	2	−2.06	−5
17	There is insufficient information and opportunities for entering job roles unrelated to my major.	−0.19	−1	1.45	4 *	−1.03	−3	−0.29	−1
18	The financial burden of obtaining certifications and preparing credentials is significant.	−1.22	−3	−1.11	−3	−0.32	0	0.01	0
19	It is difficult to choose a double or minor major due to the characteristics of my field of study.	−1.85	−4 *	1.54	4 *	−0.64	−1 *	−2.57	−5 *
20	Counselling to assess the job suitability of humanities students is necessary.	0.79	2	0.42	1	0.13	0	−0.28	−1
21	Students need more practical curricula that they can immediately apply in the workplace.	0.81	2	1.55	5	1.15	3	1.16	3
22	Schools should expand internship opportunities for humanities majors to gain practical experience.	1.11	3	1.35	3	1.65	4	1.46	4
23	Schools should further expand digital education related to the AI industry.	0	0	1.45	4 *	0.42	1	0.5	2
24	Social employment support systems, like the national employment support system, are complex and hard to access.	−0.55	−2	−0.58	−2	−0.46	−1	−1.17	−4
25	Humanities majors need more in-depth career counselling within schools for employment support.	0.29	1	−0.25	−1	−0.16	0	−0.58	−1
26	There should be more development of jobs that require humanities literacy.	0.36	1	0.18	0	−0.95	−2 *	0.67	2
27	Humanities majors need institutional support to learn new skills such as information technology (IT).	0.16	0 *	0.97	3	1.2	4	0.77	2
28	Humanities majors need information on various certifications and acquisition processes.	−0.32	−1	0.66	1	−0.4	−1	0.25	0
29	The high demand for technical skills and credentials during recruitment is burdensome.	0.21	0	−0.42	−1	1.14	3	1.71	5
30	Employment competency programmes for humanities majors are insufficient.	−0.48	−1	−0.24	0	−1.18	−4	−1.11	−3
31	Humanities majors require various credentials.	0.55	1	−0.24	0 *	0.86	2	1.42	4
32	Mentoring that connects humanities students to practical job experiences is necessary.	1.65	5	1.62	5	0.62	2	0.34	1
33	Humanities majors lack a direct link to job roles in companies.	−0.06	−1	−1.04	−3	0.21	1	−0.61	−2
34	Humanities majors find it difficult to secure competitiveness in the employment process.	0.55	1	−0.56	−1 *	0.99	2	0.18	0
35	The prejudice and social perception of humanities majors need improvement.	1.63	5 *	−0.18	0	0.01	0	0.28	0
36	There is a lack of practical information (company details, employment reviews) during job searching.	−0.72	−2	0.87	2 *	−1.03	−3	−0.67	−2
37	It is difficult to identify personal strengths or skills, causing uncertainty in career choices.	1.24	4 *	−1.45	−4	−1.66	−5	0.08	0 *
38	Strict graduation requirements make it hard to focus on job preparation.	−2.11	−5 *	−1.41	−4	2.26	5 *	−1.16	−4
39	Uncertainty about post-graduation career paths causes anxiety.	1.29	4	−1.7	−5	−1.42	−4	1.27	3
40	I feel burdened by the need to consider additional degree programmes to enhance employment prospects.	−1.2	−3	−0.28	−1	−0.99	−3	1.46	5 *

Note: * *p* < 0.05.

**Table 2:** Eigenvalues and explanatory variables of the four types.

Factor	Factor I	Factor II	Factor III	Factor IV
Eigenvalues	8.82696567	4.44859718	2.3917349	1.90183949
%Variance	29	15	8	6
%Cumulative	29	44	52	58

**Table 3:** Correlations between types.

Type	I	II	III	IV
I	1	-	-	-
II	0.1712	1	-	-
III	0.0916	0.2534	1	-
IV	0.6305	0.1252	0.2244	1

**Table 4:** Demographic background of P sample (participants) and factor loadings by type.

Type	P Sample No.	Loading	Gender	Year/Semester	Department
I (N = 10)	P28	10	Female	4/8	Korean Language and Literature
	P25	8.36468	Female	4/8	Korean Language and Literature
	P12	7.03843	Male	4/8	English Language and Culture
	P29	6.25918	Male	4/8	Korean Language and Literature
	P3	6.24515	Female	4/8	Russian Language and Culture
	P1	5.59269	Male	4/8	Russian Language and Culture
	P7	4.73502	Male	4/8	English Language and Culture
	P30	4.69949	Male	4/8	Korean Language and Literature
	P26	4.58063	Male	4/8	Korean Language and Literature
	P6	3.92595	Female	4/8	Russian Language and Culture
II (N = 6)	P17	9.04304	Female	4/8	Early Childhood Education
	P18	8.21474	Female	4/8	Early Childhood Education
	P16	8.20413	Female	4/8	Early Childhood Education
	P14	8.03822	Female	4/8	Early Childhood Education
	P19	−7.97731	Male	Graduate (Completed)	Chinese Language and Culture
	P15	7.93374	Female	4/8	Early Childhood Education
III (N = 3)	P21	13.93644	Female	4/8	Chinese Language and Culture
	P24	6.86094	Female	4/8	Chinese Language and Culture
	P23	4.41569	Female	4/8	Chinese Language and Culture
IV (N = 11)	P2	7.85429	Female	4/8	Russian Language and Culture
	P8	7.45055	Female	4/8	English Language and Culture
	P22	7.23719	Male	4/8	Chinese Language and Culture
	P4	6.35175	Female	4/8	Russian Language and Culture
	P9	5.66564	Female	4/8	English Language and Culture
	P20	4.80717	Male	4/8	Chinese Language and Culture
	P10	4.50946	Male	4/8	English Language and Culture
	P11	4.46834	Female	4/8	English Language and Culture
	P27	4.17535	Female	Graduate (Completed)	Korean Language and Literature
	P13	−3.69359	Female	4/8	Early Childhood Education
	P5	2.51791	Female	4/8	Russian Language and Culture

## Data Availability

The original contributions presented in the study are included in the article, further inquiries can be directed to the corresponding author.
